# Fingolimod (FTY720): First approved oral therapy for multiple sclerosis

**DOI:** 10.4103/0976-500X.77118

**Published:** 2011

**Authors:** Sushil Sharma, A G Mathur, Sapna Pradhan, D B Singh, Sparsh Gupta

**Affiliations:** *Department of Pharmacology, Army College of Medical Sciences, Delhi Cantt, New Delhi, India*

## INTRODUCTION

Multiple sclerosis (MS) is a chronic and disabling disease, and after trauma, it is the second most common neurological disability to affect young and middle-aged adults.[1,2] It affects twice as many women as men, with the relapsing-remitting forms of MS the most common. It is estimated that about 2.5 million people have MS worldwide.[3] The advent of the first generation of disease-modifying drugs, which include interferon beta-1a and -1b as well as glatiramer acetate, represented an important advancement in the treatment of MS when introduced into clinical practice. However, they have to be given parenterally and require either daily or weekly injections depending on the medication. These agents are known to benefit the clinical and radiological markers of relapsing MS as seen from the reduction in the frequency and severity of exacerbations and also the number of lesions visible on MRI, respectively. However, while these agents have an immunomodulatory effect that alters the course of the disease, they do not reverse the neurological damage that occurs in MS. Fingolimod (FTY720) is a once-daily oral drug developed by switzerland based drug major Novartis Pharmaceuticals, with potential disease-modifying effects and as such may offer a significant benefit over injectable agents.[4] The US Food and Drug Administration (FDA) approved fingolimod on September 22, 2010, to reduce relapses and delay disability progression in patients with relapsing forms of MS.[5]

## MECHANISM OF ACTION

Patients with MS display a wide range of symptoms that primarily arise from demyelination in the central nervous system (CNS), which includes the brain, spinal cord, and optic nerves. The destruction of the protective myelin sheath that surrounds nerve cells and axonal damage are thought to be due to the autodestructive effects of inflammatory T cells.[6,7] Fingolimod acts as a modulator of the sphingosine-1-phosphate receptor (S1P-R) on the surface of thymocytes and lymphocytes, and sequestrates them in lymph nodes away from the CNS.[8,9] This reduces the number of inflammatory cells in the circulation and CNS, and in doing so reduces their potential to damage the myelin sheath surrounding axons in MS nerve cells.[10,11] Data from preclinical studies indicate that fingolimod may mediate its effects through a variety of mechanisms, some of which may be independent of lymphocyte depletion and enhanced myelination and axonal protection following the oral administration of fingolimod have been reported.[12–14]

## PHARMACOKINETICS

Fingolimod is a close structural analog of sphingosine and after oral administration, it is phosphorylated by sphingosine kinase to form the active phosphate moiety which subsequently binds to the S1P-R.[15] Fingolimod is predominantly hydroxylated by cytochrome CYP4F2. Ketoconazole has been shown in *in vitro* experiments to significantly inhibit the oxidative metabolism of fingolimod by human liver microsomes and also by recombinant CYP4F2.[16] This interaction should be kept in mind while adding ketoconazole to a fingolimod regimen. The Tmax of fingolimod is 12–16 hrs and the bioavailability after an oral dose is 93%. No interaction with food has been seen.

## CLINICAL TRIALS

The efficacy and safety of fingolimod have been evaluated by two large Phase III. These were the FREEDOMS (FTY720 Research Evaluating Effects of Daily Oral therapy in Multiple Sclerosis) and TRANSFORMS (TRial Assessing injectable interferoN vS FTY720 Oral in RrMS) trials.

The FREEDOMS[17] trial was a 24-month, double-blind, placebo-controlled trial of 1272 patients with relapsing–remitting MS who were randomized to receive placebo or either 0.5 mg or 1.25 mg of oral fingolimod daily. After 24 months, the annualized relapse rate which was the primary endpoint was significantly reduced with both doses of fingolimod versus placebo. The risk of disability progression during 24 months and the cumulative probability of disability progression, at 3 months, were also significantly reduced with fingolimod in both doses as compared to placebo. Radiological parameters, including new or enlarged lesions on T2-weighted images, gadolinium-enhancing lesions, and brain volume loss as seen on MRI also showed a superior response with both doses of fingolimod as compared to placebo.

In the TRANSFORMS[18] trial, 1292 patients with relapsing–remitting MS were randomized to receive oral fingolimod, in the doses of 0.5/1.25 mg/day, or interferon beta-1a, 30 μg, given intramuscularly once a week. To protect the blinding, all patients in the fingolimod group received placebo injections once a week, while those randomized to interferon beta-1a received a placebo pill once a day. The primary endpoint, namely, the annualized relapse rate, showed a significant reduction in both fingolimod groups as compared with the interferon beta-1a group. Disease activity as seen on MRI was also reduced in both fingolimod groups versus the interferon beta-1a group.

## ADVERSE EFFECTS

The common adverse effects seen with fingolimod are headache, influenza-like illness, nasopharyngitis, and fatigue.[19] A sudden reduction in the heart rate most commonly seen within 6 h of the first dose of fingolimod has been reported in the trials.[17,18] Patients should be monitored for bradycardia and atrioventricular blockade and a baseline ECG is recommended when starting fingolimod therapy especially in high-risk individuals. Macular edema has also been seen and an ophthalmologic evaluation is recommended for those taking the drug. An increase in the incidence of skin cancer and liver enzymes has also been found.[17–19] Treatment with fingolimod is also associated with an increased risk for infection. Two deaths were seen during the TRANSFORMS study, both of which were in the group receiving fingolimod, 1.25 mg/day: one patient succumbed to disseminated primary varicella zoster and the other died because of herpes simplex encephalitis.[18]

## CURRENT STATUS

Fingolimod is the first oral drug approved by the US FDA for the treatment of MS in the dose of 0.5 mg. As of now, experts do not recommend a change of drug in patients who are well stabilized on their existing interferon therapy due to the serious nature of adverse effects of fingolimod including severe bradycardia, macular edema, and life-threatening infections.[20] Nevertheless, an oral therapy is a very welcome addition to the armamentarium of the neurophysicians working on MS who will be expected to exercise their clinical judgment in consultation with their patients so as to decide the most appropriate therapy for their condition.

## CONCLUSION

Fingolimod is a breakthrough in the treatment of multiple sclerosis as it is the first oral agent available for this disorder. Yet this drug is not the first choice as it has serious adverse effect profile. Fingolimod has opened new vistas in the therapy of multiple sclerosis and a search for similar drugs with better safety profile is already initiated. This area in the drug discovery is likely to witness intense activity in the near future.
